# Leukocyte count affects expression of reference genes in canine whole blood samples

**DOI:** 10.1186/1756-0500-4-36

**Published:** 2011-02-09

**Authors:** Christine J Piek, Bas Brinkhof, Jan Rothuizen, Aldo Dekker, Louis C Penning

**Affiliations:** 1 Department of Clinical Sciences of Companion Animals, Utrecht, Utrecht University, PO Box 80154, 3508 TD Utrecht, The Netherlands; 2 Central Institute for Animal Disease Control, P.O. Box 2004, 8203 AA Lelystad, The Netherlands

## Abstract

**Background:**

The dog is frequently used as a model for hematologic human diseases. In this study the suitability of nine potential reference genes for quantitative RT-PCR studies in canine whole blood was investigated.

**Findings:**

The expression of these genes was measured in whole blood samples of 263 individual dogs, representing 73 different breeds and a group of 40 mixed breed dogs, categorized into healthy dogs and dogs with internal and hematological diseases, and dogs that underwent a surgical procedure. GeNorm analysis revealed that a combination of 5 to 6 of the most stably expressed genes constituted a stable normalizing factor. Evaluation of the expression revealed different ranking of reference genes in Normfinder and GeNorm. The disease category and the white blood cell count significantly affected reference gene expression.

**Conclusions:**

The discrepancy between the ranking of reference genes in this study by Normfinder and Genorm can be explained by differences between the experimental groups such as "disease category" and "WBC count". This stresses the importance of assessing the expression stability of potential reference genes for gene experiments in canine whole blood anew for each specific experimental condition.

## Findings

### Context

The dog is frequently used as an experimental model for hematologic human diseases[1]. The use of dogs can be explained by the fact that the dog offers a variety of spontaneous and experimental models of hematologic diseases. Recent examples are the use of canine hemophilia A [2] and B models [3,4], and the Canine Leukocyte Adhesion Deficiency model (CLAD) [5,6] in gene therapy experiments [2-8], and pharmacological experiments in leukopenic dogs [7] and in dogs with CLAD [8]. The larger size of dogs compared to small rodent models allows similar surgical procedures in humans as in dogs, and permits in most cases adequate acquisition of diagnostic samples. The dog has been a longstanding model for bone marrow and more recently for stem cell transplantations [9,10]. Anticoagulant therapy has been tested extensively in canine cardiac surgery models [11,12]. Also the pathogenesis and therapy of acquired disorders of hemostasis such as disseminated intravascular coagulation[13], thrombosis [14,15], and hemolytic uremic syndrome [16] have been investigated in canine models.

A disadvantage of the canine model compared to human or small rodent models is the limited availability of antibodies against canine intra- and extracellular proteins such as CD markers. At least 350 CD markers are defined in humans [17,18], while in the first and to date only workshop on canine leukocyte antigens only 127 antibodies were investigated [19]. A more recent study tested cross species reactivity with commercially available anti human CD molecules against canine leukocytes, erythrocytes and platelets and identified only a limited number of 51 cross reacting mAbs [20]. In contrast to the limited knowledge of canine CD markers, the canine genome has been sequenced in total [21]. Therefore most molecular tools can be readily applied in dog research. Real-time quantitative reverse transcriptase PCR (Q-PCR) offers an accurate and sensitive alternative to quantification of gene expression [22] and for that reason is well suited to study biological processes and has also many practical clinical applications. Q-PCR has already been shown to be a valuable adjunct in immunophenotyping and the quantification of residual disease in leukemia [23-26].

Multiple variables need to be controlled when performing a Q-PCR, such as the quality of RNA after isolation, the input amount and quality of mRNA and cDNA reaction efficacy, efficiency of the enzymatic reactions, and cell to cell variability in translational activity. One of the solutions to control for the internal variation that affect the outcome of the Q-PCR reaction is the use of reference genes as an internal standard [22,27]. Reference genes are selected based on the supposition that their expression is stable in all cells regardless of the tissue or individual [28]. It has been proven, however, that many genes essential for basic cellular mechanisms and hitherto thought to have a stable expression throughout the organism actually did not comply with this assumption [29-35]. Therefore, it is essential that the assumption of stable expression of potential reference genes is verified for each experimental set up [28,36-38].

In this study we investigated the suitability of nine frequently used reference genes in Q-PCR for the use as reference genes in a quantitative real-time PCR in canine whole blood and the influence of dog breed, sex, disease category and disease duration on the Cq of these genes was assessed.

## Methods

### Blood sample collection

Between September 2007 and October 2008 canine blood samples (n = 263) were taken from dogs submitted to the intensive care unit of the small animal hospital of the Veterinary Faculty of the Utrecht University (The Netherlands) from healthy control dogs (n = 6; group A) and dogs categorized into three disease groups. Group B (n = 85) had surgery within the preceding 24 hours, group C (n = 107) were dogs with miscellaneous internal diseases, and group D (n = 65) had hematologic disease (disseminated intravascular coagulation (n = 27), systemic inflammatory disease (n = 24), and immunemediated hemolysis (n = 14).

The 263 dogs represented 73 different breeds and a group of mixed breed dogs (n = 40). Breeds that were represented by at least 5 dogs were the Labrador retriever (n = 30), Golden retriever (n = 18), Jack Russell terrier (n = 10), (Bordeaux dog (n = 9), Dachshund n = 9), Boxer (n = 7), German shepherd and German pointer (n = 6), and the Bernese mountain dog, Beagle, English Cocker spaniel, and the Bearded collie were all represented by 5 dogs. There were 42 female dogs, 91 castrated female dogs, 78 males, and 47 castrated male dogs. Of 3 dogs the sex was not noted in the file. The mean age of the dogs was 6.5 years (range 12 weeks to 14 years, SD 3.5 years).

Two milliliters of EDTA-anticoagulated blood were collected from each dog on the day of admittance and during the period the dog was hospitalized consecutive samples were taken at least 24 hours apart.

From 99 of the dogs, a second sample was available (37 of group B, 30 of group C, 32 of group D), and in, respectively, 34 dogs a third (10 of group B, 6 of group C, 18 of group D), and in 13 dogs a fourth sample (4 of group B, 3 of group C, 6 of group D) was available.

All procedures were approved by and performed according to the ethical committee as required under Dutch legislation.

### RNA isolation and cDNA synthesis

In view of the large sample number but small samples size, the RT-reaction was performed only once. The MIQE guidelines, however, suggest to perform it twice [39,40]. From each dog duplo samples were prepared by mixing 0.5 ml EDTA-anticoagulated blood with 1.3 ml RNA*later *(Ambion, Applied Biosystems, Foster City, California, USA). Samples were stored at -20°C. Total RNA was extracted from the samples using the RiboPure™-Blood kit reagent (Ambion, Applied Biosystems, Foster City, USA) according to the manufacturer's instructions including a DNAse treatment to destroy contaminating genomic DNA and minimize the effect of pseudogenes. The RNA concentration was determined spectrophotometrically by the NANOdrop 1000 Isogen Life Science, IJsselstein, The Netherlands). Bio-Rad iScript, containing both oligodT and random hexamer primers, was used to synthesize cDNA from 1 μg of total RNA according to the manufacturer's instructions (iSCRIPT, Bio-Rad, Veenendaal, The Netherlands).

### Primer design and testing

The selection and testing of candidate reference genes was based on gene targets that have already been used in human and veterinary research, and have been previously reported [41,42]. Nine genes representing various biological processes (GAPDH, SRPR, HPRT, B2M, GUSB, HNRNPH, RPL8, RPS5, RPS19) were selected as candidate reference genes. Their full names, GenBank accession numbers, and location in the canine chromosome are given in Table 1. The primers that were used, location of these primers within the gene, and the length of the resulting amplicon are given in Table 2. Primers were developed based upon known canine sequences (Ensembl, http://www.ensembl.org and GenBank, http://www.ncbi.nih.gov/genbank/index.html). The primers were designed with Oligo Explorer 1.1 (http://www.genelink.com/tools/gl-downloads.asp). The specificity and uniqueness of each primer were verified with the Basic Local Alignment Search tool expecting return of Genbank accession numbers of candidate reference genes only (http://www.genelink.com/tools/gl-downloads.asp). All primer pairs, except for GAPDH, were intron spanning. The PCR reaction was optimized for the primers. Optimal Tm values ranged from 55°C for RPL8 to 62.5°C for RPS5 (Table 2). Amplification efficiency calculations from all standard curves were between 93.9 and 106.7%. All no template controls were negative.

**Table 1:** Abbreviations, GenBank Accession numbers, names, and chromosomal location of canine candidate reference genes evaluated.

Gene	**GenBank Accession No**.	Name	Chromosomal location in *Canis familiaris*
RPS19	XM_533657	Ribosomal protein S19	Chromosome 1
RPL8	XM_532360	Ribosomal protein L8	Chromosome 13
RPS5	XM_533568	Ribosomal protein S5	Chromosome 1
GUSB	NM_001003191	Beta-Glucuronidase	Chromosome 6
B2M	XM_535458	Beta-2-microglobulin	Chromosome 30
HNRNPH	XM_538576	Heterogeneous nuclear ribonucleoprotein H	Chromosome 11
HPRT	AY283372	Hypoxanthine phosphoribosyltransferase 1	Chromosome X
GAPDH	NM_001003142	Glyceraldehyde-3-phosphate dehydrogenase	Chromosome X
SRPR	X03184	Signal recognition particle receptor	Chromosome 5

**Table 2:** Primer sequences, exon locations, amplicon size, and optimal melting temperature of canine candidate reference genes.

Gene	Forward 5' →3'*	Exon(s)	Reverse 5' → 3'	Exon(s)	Product length (bp)	**T**_**m **_**(°C)**
RPS19	CCTTCCTCAAAAA/GTCTGGG	2/3	GTTCTCATCGTAGGGAGCAAG	3	95	61.0
RPL8	CCATGAAT/CCTGTGGAGC	4/5	GTAGAGGGTTTGCCGATG	5	64	55.0
RPS5	TCACTGGTGAG/AACCCCCT	2/3	CCTGATTCACACGGCGTAG	3	141	62.5
GUSB	AGACGCTTCCAA/GTACCCC	3/4	AGGTGTGGTGTAGAGGAGCAC	4	103	62.0
B2M	TCCTCATCCTCCTCGCT	1	TTCTCTGCTGGGTGTCG	2	85^†^	61.2
HNRNPH	CTCACTATGATCCACCACG	5	TAGCCTCCATAAC/CTCCAC	5/6	151	61.2
HPRT	AG/CTTGCTGGTGAAAAGGAC	5/6	TTATAGTCAAGGGCATATCC	7	114^‡^	56.0
GAPDH	TGTCCCCACCCCCAATGTATC	2	CTCCGATGCCTGCTTCACTACCTT	2	100	58.0
SRPR	GCTTCAGGATCTGGACTGC	5/6	GTTCCCTTGGTAGCACTGG	6	81	61.2

* If a primer is located on two exons, the junctions are shown with a dividing forward slash (/).

^† ^Genomic product size would be approximately 3.6 kb.

^‡ ^Genomic product size would be approximately 300 bp.

### Quantitative PCR

Q-PCR was done with the DNA-binding SYBR green using the BioRad iCycler MyiQ Real-Time PCR Detection System (BioRad, Hertfordshire, United Kingdom) according to the manufacturer's instructions. Primers (Eurogentec, Maastricht, The Netherlands) had a final concentration of 400 nM each. One microliter of cDNA was used per Q-PCR reaction. Optimal *T*_m _was determined previously [41,42]. Reactions with a *T*_m _less than 58°C started with 5 min at 95°C, followed by 40 cycles of 20 s at 95°C, 30 s at *T*_m_, and 30 s at 72°C. This reaction was continued by a melting curve, stepwise increasing temperature each 15 s by 0.5°C, ranging from 60 to 95°C. In case the *T*_m _was 58°C or higher, the elongation step at 72°C was omitted and *T*_m _remained 30 s. Analysis of Q-PCR results were performed with iQ™5 software (Biorad, Veenendaal, The Netherlands) based on the mean Cq obtained from the duplo of each Q-PCR reaction.

### Analysis gene expression

Firstly, the influence of experimental condition such as disease category and duration, sex, leukocyte count on potential reference gene expression were determined. For each potential reference gene a comparison of the mean Cq values obtained at the first sampling for the disease groups A, B, C, and D, and sex was performed using the ANOVA. To determine if the differences in Cq's for the nine potential reference genes were due to changes in expression levels over time an ANOVA was used. Using a forward selection process, two explanatory variables, "dog" and "sample number", were introduced as factors in the ANOVA. The result variable was the observed Cq value. The resulting models were compared using the likelihood ratio test.

The mean Cq values for dogs with a leukocyte count within the reference range (4.5 - 14.6 *10^9^/l) were compared to mean Cq's of dogs with a leukocyte count above 30 *10^9^/l, which can be considered a clinically relevant leukocytosis. If a significant difference was observed, a pair wise comparison was made using the T-test with Holmes correction for multiple comparisons. Secondly, a linear mixed effects model was used to assess the significance as well as the magnitude of the effect of leukocyte count on Cq per dog, with the mean Cq as response variable, the natural logarithm of the "leukocyte count" as explanatory variable, and the "dog" as random effect. Similarly, a linear mixed effects model was used to determine if the leukocytes count changed over time per dog. An ANOVA was used to compare the leukocyte counts in the disease groups A, B, C and D. A linear model was used to examine the relationship of the Cq with the variables "disease category" and the natural logarithm of the "leukocyte count".

All statistical analyses were performed in R (http://www.r-project.org). P below 0.05 was considered significant in all analyses.

To determine the ranking of best performing reference genes in whole blood the stability of expression of the candidate reference genes was calculated using the GeNorm [27] and Normfinder[43] algorithm software. The gene expression stability calculations in this study were performed on the first sample that was taken when the dog entered the study.

In Genorm the expression ratio for each pair of candidate reference genes is calculated for the data array of all samples and log_2_-transformed. "M" is the arithmetic mean of the pair wise variation measured as the standard deviation of thus obtained values. A low "M" indicates little variation in expression of the two genes. Then the optimal number of control genes for normalisation is determined. Firstly, the normalisation factor is calculated based on the two reference genes with the lowest "M"-values. Secondly, the contribution of an additional candidate reference gene to the variance of the normalisation factor ratios is calculated by the stepwise introduction of the reference genes following the earlier established ranking order of their "M"-values.

Shortly, Normfinder makes use of a mathematical model to describe the expression values measured by RT-PCR, separate analysis of the sample subgroups, and estimation of both the intra- and the intergroup expression variation, and finally calculates a candidate gene "Stability Value."

## Results

### Expression of candidate reference genes

**Figure 1:**
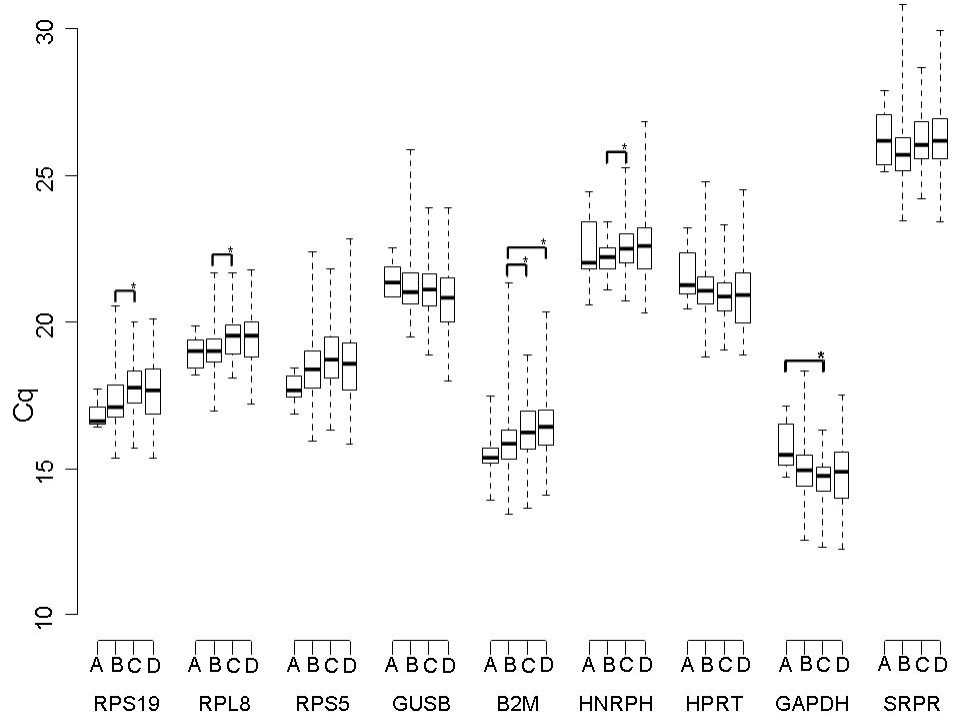
**Real-time PCR cycle threshold numbers (Cq values) for nine potential reference genes in 4 disease categories (n = 263) **. Real-time PCR cycle threshold numbers (Cq values) are plotted for nine potential reference genes. Group A included 6 healthy dogs, group B 85 dogs within 24 hours after a surgical procedure, group C 107 dogs with miscellaneous internal diseases, and group D 65 dogs with hematologic diseases. Statistically significant differences between mean Cq of the disease categories are depicted. The boxes represent the two middle quartiles with medians. Whiskers delineate the range.

The range and median Cq values of the first sample that was taken in the dogs in the disease groups A, B, C and D (described above) are depicted in Figure 1. There was a significant difference between the mean Cq's measured in groups B and C for RPL8, RPS19, B2M and HNRNPH, the differences being 0.35, 0.39, 0.44, and 0.35 Cq, respectively. The difference between groups B and D for B2M was 0.51 Cq, and between A and C for GAPDH it was 1.1 Cq (Figure 1). "Sample number" did not significantly determine the Cq, except for SRPR (p = 0.013), nor did "sex" and "breed".

Next the leukocyte count was examined. The leukocyte counts of disease group A was within the reference range (median 8.6, range 6.6 - 12.5*10^9^/l). The leukocyte counts of disease groups B (median 15.9, range 3.8 - 107.8*10^9^/l) and C (median 16.8, range 2.1 - 44.6*10^9^/l) were statistically significant from group D (median 22.6, range 4.8 - 175.9*10^9^/l) (P = 1.9*10^-7 ^and 7.8*10^-6 ^respectively). The linear mixed effects model revealed that "leukocyte count" did not significantly change between sequential samples taken during the course of the disease.

The linear mixed effects model that included only "leukocyte count" as explanatory variable for the Cq was not significant for SRPR, HNRNPH, and GUSB. The other 6 potential reference genes (B2M, RPL8, RPS19, RPS5, GAPDH, and HPRT) had significant changes in Cq, ranging from -0.87 to 1.28 for an a ten fold increase in leukocyte count. A significant difference between the Cq's of dogs with a leukocyte count within the reference range and dogs with a leukocyte count above 30 *10^9^/l was found for RPS19, RPL8, RPS5, B2M, and HPRT. Additionally, in this analysis, GAPDH, was identified as the fourth of the nine reference genes that was not significantly influenced by leukocyte count (Table 3).

**Table 3:** Relation of Cq and white blood cell count.

Gene	Cq change for 10 fold increase in WBC count	p	WBC within reference range		WBC > 30*10^**9**^**/l**		Difference	p
								
		
			Mean Cq	SD	Mean Cq	SD		
RPS19	0.92	0	17.35	0.82	17.90	1.31	0.56	0.002
RPL8	0.52	0.006	19.20	0.67	19.52	1.14	0.32	0.026
RPS5	1.06	0	18.30	0.86	19.01	1.61	0.71	0.0009
**GUSB**	**- 0.07**	**0.75**	21.09	0.97	21.08	1.47	**-0.01**	**0.93**
B2M	1.28	0	15.85	0.86	16.58	1.74	0.73	0.0006
**HNRNPH**	**- 0.09**	**0.68**	22.40	0.86	22.20	1.02	**-0.20**	**0.19**
HPRT	- 0.87	0	21.14	0.93	20.71	1.03	-0.43	0.01
**GAPDH**	- 0.51	0.023	14.87	0.97	14.80	0.96	**-0.07**	**0.38**
**SRPR**	**0.10**	**0.70**	18.30	1.09	19.01	1.51	**0.03**	**0.91**

• WBC = White Blood Cell Count

Change in Cq is given for a ten fold increase in WBC count, mean Cq and SD for dogs with WBC count in the reference range and for dogs with a leukocytosis with a WBC > 30*10^9^/l.

The linear model that included both "leukocyte count" and the "disease category" as explanatory variables for the Cq was statistically significant for both RPS5 and B2M. "Disease category" was the statistically significant factor determining Cq in the case of SRPR, HNRNPH, GUSB and GAPDH and "leukocyte count" in the case of RPS19, RPL8, and HPRT.

**Figure 2:**
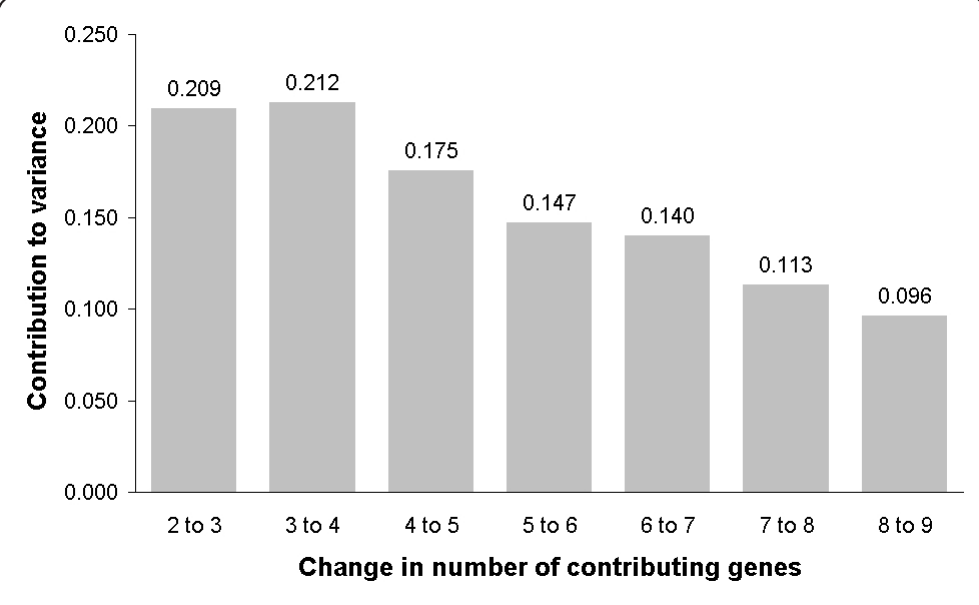
**Pairwise variations between 2 sequential normalization factors including an increasing number of potential reference genes **. To determine the optimal number of reference genes, first the geometric mean of the expression of the previously ranked genes was calculated and then pair wise variations between sequential normalisation factors were calculated. Using the cut-off recommended by GeNorm of 0.15 the optimal number of reference genes for the data set in this study would be at least 5.

In order to identify the genes that had the least variable expression, expression stability was evaluated using GeNorm and Normfinder software analysis. The pair wise variation between the normalisation factors calculated by GeNorm steadily decreased after inclusion of the fourth additional reference gene and falls below the cut-off of 0.15 that is suggested by the GeNorm programme after adding the fifth gene [27] (Figure 2). The ranking of the potential reference genes by GeNorm and Normfinder is given in Table 4.

**Table 4:** Ranking of potential reference genes according to their expression stability by GeNorm and Normfinder.

GeNorm	Stability measure*	Normfinder	Stability Value*	Normfinder	Stability Value*
	M	Disease category**		WBC quartiles***	
RPL8 and RPS19	0.55	RPL8	0.16	RPL8	0.09
		HNRNPH	0.17	HNRNPH	0.14
RPS5	0.64	SRPR	0.19	GUSB	0.14
GUSB	0.78	GUSB	0.26	SRPR	0.16
B2M	0.87	HPRT	0.26	GAPDH	0.17
HNRNPH	0.93	RSP19	0.26	RSP19	0.17
HPRT	0.99	B2M	0.28	RPS5	0.21
GAPDH	1.01	RPS5	0.32	HPRT	0.21
SRPR	1.03	GAPDH	0.35	B2M	0.21

*Genes are ranked according to decreasing expression stability.

## Discussion

Studying gene expression by the sensitive, specific, and accurate technique of quantitative RT-PCR has become increasingly important in biomedical research. The goal of this study was to select reference genes that can be used as a normaliser when studying gene transcription in canine blood cells. Nine genes that are either conventionally used as reference genes or were shown to have a stable expression in hematopoietic cells or whole blood were chosen as potential candidate reference genes in this study [36,41,42,44,45] (Table 1). Even genes that regulate basic cellular tasks have been shown to be regulated [29-35,46]. To exclude that the expression of the potential reference genes was influenced by the experimental conditions in our study we investigated the effect of several parameters such as disease category, disease duration, and leukocyte count. Additionally, two software algorithms, respectively, Normfinder [43] and GeNorm [27], were used to calculate gene expression stability and help select the combination of reference genes that provides the most stable normalizer for a specific experimental situation.

Whole blood RNA originating from all cells present in the peripheral blood, as opposed to RNA derived from a cell sorting procedure, was used for the reverse transcriptase reaction in this study. To correct for the leukocyte count reaction was performed on a fixed amount of starting RNA. The influence of a disproportionate increase of a subset of the leukocytes on reference gene expression is not countered by this. This disadvantage has to be weighed against the advantage of being able to investigate simultaneously the expression of multiple genes originating from distinct cell types. And, additionally, against the fact that cell sorting procedures have been shown to affect gene expression. Five to nine fold up regulation of cytokine expressions were seen after density gradient separation of leukocytes [47].

Several conditions that might effect gene expression were examined in this study. Figure 1 reveals significant increases in Cq between the disease groups for RPS19, RPL8, B2M, HNRNPH, and GAPDH, The maximum increase is seen in case of B2M between groups B and D (0.51 Cq). These differences between the disease groups can be contributed mainly to the disease condition as opposed to disease duration since the Cq was not significantly different between the sequential samples taken during the disease period in a subset of the dogs. The leukocyte count gradually increases comparing the disease groups, revealing a significant difference between group B (median leukocyte count 15.9*10^9^/l) versus C (median leukocyte count 16.8*10^9^/l) and D (median leukocyte count 22.6*10^9^/l), respectively. The changes in Cq associated with leukocyte count had a similar direction as the Cq changes in the disease categories (Figure 1). This suggested that "leukocyte count" might a major factor explaining the directional change in Cq. The linear model examining the influence of "disease category" and "leukocyte count" revealed that this was the case for RPS19 and RPL8. RPS5 and B2M were best explained by the linear model containing both parameters, however. The reference genes that were not significantly influenced by the WBC count were GUSB, HNRNPH and SRPR (Table 3).

B2M has shown a highly variable expression in several tissues other than whole blood [41,42,44,48] but had a stable expression in one study where human leukocytes from 13 healthy donors were examined [27]. B2M also had stable expression in a large study where 526 human whole blood samples representing healthy individuals and 6 disease groups [49]. The influence of leukocyte count on B2M expression was not examined in both these studies. B2M encodes for beta-2-microglobulin which is part of the canine MHC I molecule and abundantly expressed on hematopoietic cells. The decrease in B2M expression associated with increases in leukocyte count in this study might reflect both a decrease in induced expression, or a shift in leukocyte subsets displaying different MHC class I receptor densities.

The selection of one, or a set of, potential reference genes for a future experiment depends besides practical points such as available sample sizes and costs mainly on stability of expression in the experimental samples. In this study we evaluated the expression stability with Normfinder and GeNorm. Both software algorithms are frequently used and freely available but have a different working rationale. Normfinder selects out of a set of potential reference genes one single, or the pair of, best performing reference genes that show the least variation between and within experimental groups. The focus on the detection of directional changes in reference gene expression due to differences between the experimental groups is the major difference with GeNorm that focuses on pair wise comparisons of reference gene expression in the experimental samples and is therefore less apt to identify coregulated genes [50]. Since WBC count and disease category had a statistically significant effect on potential reference gene expression, it is not surprising that the ranking provided by Normfinder and GeNorm differed. Among the genes ranked highest by Normfinder were the genes that were not significantly influenced by the WBC count (GUSB, HNRNPH and SRPR, Table 3).

Contrastingly, GeNorm ranked RPS8, RPS19, and RPS5 highest. Similarly, RPL8 had the best Stability Value in Normfinder, but both RSP19 and RSP5 were ranked at the low end of the list (Table 4). An explanation might be that these three genes are all coding for ribosomal proteins which are likely to be coregulated. Despite the fact that they have less variation in expression as pointed out by GeNorm, the directional difference in expression of these coregulated reference genes, will potentially decrease the sensitivity of detecting changes in expression of the genes of interest in an experiment [51].

The discrepancy between the ranking of reference genes in this study by Normfinder and Genorm can be explained by differences between the experimental groups such as "disease category" and "WBC count". These results reveal that experimental conditions can result in unforeseen group wise up regulation or down regulation of reference genes that otherwise may have a stable expression when the whole dataset is considered. Minor group specific directional changes in reference gene expression might obscure changes in candidate gene expression between groups. The results of this study emphasize that it is prudent to assess each new data set specifically for changes in reference gene expression due to the experimental conditions even when reference genes are chosen that were previously shown to have a stable expression.

## List of abbreviations

B2M: beta-2-Microglobulin; GAPDH: Glyceraldehyde-3-phosphate dehydrogenase; GUSB: beta-Glucuronidase; HNRNPH: Heterogeneous nuclear ribonucleoprotein H; HPRT: Hypoxanthine phosphoribosyltransferase; RPL8: Ribosomal protein L8; RPS5: Ribosomal protein S5; RPS19: Ribosomal protein S19; SRPR: Signal recognition particle receptor.

## Competing interests

The authors declare that they have no competing interests.

## Authors' contributions

CJP participated in the experimental design, collected blood samples and drafted the manuscript. BB carried out the molecular analysis. JR was involved in the experimental design and sample collection. AD carried out the statistical analysis. LCP participated in the experimental design and coordination and drafted the manuscript.

All authors read and approved the final manuscript.
